# The Impact of Maternal Diabetes and Hypothyroidism on Signaling Pathway Activation and Gene Expression in Fetal Mesenchymal Stem Cells

**DOI:** 10.3390/biomedicines14020436

**Published:** 2026-02-14

**Authors:** Dominika Przywara, Wiktor Babiuch, Alicja Petniak, Bartosz Piszcz, Arkadiusz Krzyżanowski, Adrianna Kondracka, Janusz Kocki, Paulina Gil-Kulik

**Affiliations:** 1 Department of Clinical Genetics, Medical University of Lublin, 11 Radziwillowska Str., 20-080 Lublin, Poland; dprzywara17@gmail.com (D.P.); alicja.petniak@umlub.pl (A.P.); janusz.kocki@umlub.pl (J.K.); 2 Doctoral School, Medical University of Lublin, 20-093 Lublin, Poland; piszczb@gmail.com; 3 Student Scientific Society of Clinical Genetics, Medical University of Lublin, 11 Radziwillowska Str., 20-080 Lublin, Poland; babiuchwik@gmail.com; 4 Department of Obstetrics and Pathology of Pregnancy, Medical University of Lublin, 11 Staszica Str., 20-081 Lublin, Poland; arkadiusz.krzyzanowski@umlub.pl (A.K.); adrianna.kondracka@umlub.pl (A.K.)

**Keywords:** mesenchymal stem cells, umbilical cord, signaling pathways, gene expression, diabetes mellitus, hypothyroidism, pregnancy

## Abstract

**Background**: Mesenchymal stem cells (MSCs) exhibit a high capacity for differentiation, possess anti-inflammatory and proangiogenic properties, and stimulate the growth and proliferation of neighboring cells. MSCs are a promising tool in regenerative medicine. However, the molecular mechanisms underlying the properties of these cells are not yet fully understood. Gene expression in MSCs influences their characteristics and differentiation potential. Therefore, it is essential to investigate factors affecting gene expression as well as those activating signaling pathways, which will enable more effective and individualized applications of MSCs. In this study, we aimed to identify signaling pathways involved in gene expression in umbilical cord-derived MSCs (UC-MSCs) that may be altered by maternal diabetes and hypothyroidism during pregnancy. **Methods**: The research material consisted of UC-MSCs. Samples obtained from nine participants were analyzed. UC-MSCs were isolated and cultured, and RNA was extracted. The isolated RNA was used for microarray-based gene expression analysis. Subsequently, pathway enrichment analysis was performed to identify the signaling pathways involved. **Results**: In the diabetes group, 340 genes (0.71%) were upregulated, while 268 genes (0.56%) were downregulated compared with UC-MSCs from the control group. In the diabetes group, the most compact module was composed of proteins associated with WNT/planar cell polarity (WNT/PCP) signaling. The second module included genes related to smooth muscle activity. In the hypothyroidism group, an association was identified between the extracellular matrix organization pathways (GO:0030198) and the extracellular structure organization (GO:0043062) pathways. Moreover, in this group, increased expression of *MMP1*, *MMP10*, and *GREM1* was observed. **Conclusions**: In summary, our study demonstrated the impact of diabetes and hypothyroidism on gene expression in UC-MSCs. We also observed the activation of distinct signaling pathways depending on the presence of these conditions. However, this work represents a preliminary screening, and the results should be validated by PCR in a larger cohort.

## 1. Introduction

The umbilical cord (UC), primarily composed of Wharton’s jelly and fetal blood vessels, serves as a critical interface between the mother and fetus, reflecting the intrauterine environment’s impact on fetal development [1,2,3,4].

The main tissue constituting the UC is Wharton’s jelly. It is a type of connective tissue composed of MSCs [5]. MSCs exhibit a high capacity for differentiation. Moreover, they possess anti-inflammatory and proangiogenic properties and stimulate the growth and proliferation of neighboring cells [6]. Considering the properties of MSCs and the fact that umbilical cord cells represent fetal cells, it can be inferred that UC-MSCs, to some extent, influence fetal development.

Moreover, UC-MSCs are a promising tool in regenerative medicine, as their proangiogenic properties and ability to stimulate cell growth may accelerate tissue regeneration and even contribute to tissue reconstruction due to their broad differentiation potential [6]. UC-MSCs are a cornerstone of regenerative medicine due to their broad differentiation potential and immunomodulatory properties, with ongoing clinical trials targeting various metabolic and degenerative disorders [6,7,8,9,10,11,12,13]. The application of MSCs is not limited to a single specific type of disorder but appears to be relatively universal. Depending on the underlying pathomechanism of the disease, different properties of MSCs may be particularly beneficial. For neurodegenerative disorders, neuroprotective and anti-apoptotic effects are of primary interest, whereas in autoimmune conditions, their anti-inflammatory properties are especially valuable [14,15].

However, before UC-MSCs can be used for therapeutic or diagnostic purposes, a deeper understanding of their molecular mechanisms is required. It is essential to investigate factors that affect gene expression as well as those that activate signaling pathways. This will enable more effective and individualized applications of MSCs. The mere presence of a gene does not necessarily indicate its functional significance. Rather, it is the expression of individual genes that determines cellular properties and directs cells to perform specific functions through the proteins synthesized during translation [16]. However, the transcriptome does not always correlate with protein levels [17]. Between the gene and the protein, numerous molecules and regulatory mechanisms control gene expression. These include, among others, microRNAs and epigenetic modifications, which may be influenced by external conditions. Understanding these processes will facilitate the more effective use of UC-MSCs and provide insights into possible strategies for modifying UC-MSC culture conditions to induce desired cellular properties.

Another aspect that complicates the understanding of UC-MSC biology is the relationship between the mother and the fetus. We cannot examine the molecular processes occurring in fetal cells without taking into account the processes taking place in maternal cells. This phenomenon aligns with the Developmental Origins of Health and Disease (DOHaD) hypothesis, suggesting that maternal lifestyle and health status during pregnancy have lasting genetic and epigenetic consequences for the offspring. For instance, smoking alters the expression of genes responsible for fetal growth, whereas alcohol consumption modifies the methylation levels of genes involved in the child’s immunity [18]. Additionally, in previous studies, we demonstrated that the mother’s health status affects gene expression in the child and, in this way, influences the anti-inflammatory properties of their cells [19]. In this study, we aim to identify the signaling pathways involved in the gene expression of UC-MSCs that are altered as a result of maternal diabetes and hypothyroidism during pregnancy.

Endocrine disorders most commonly occurring during pregnancy include diabetes and hypothyroidism [20]. Hypothyroidism affects up to 5% of pregnant women and is frequently associated with obstetric complications, such as preterm birth, pregnancy loss, low Apgar scores, and hypertension [21]. Moreover, numerous studies indicate that hypothyroidism may increase the risk of developing gestational diabetes [20,22]. Diabetes is also associated with multiple complications, including neonatal complications such as hypoglycemia and jaundice [23]. In addition, our previous studies indicate that these conditions influence gene expression in MSCs [19,24,25].

While the clinical impact of maternal diabetes and hypothyroidism is well-documented, the specific molecular crosstalk between these endocrine disorders and the UC-MSC signaling networks remains poorly understood. Most existing studies focus on single-gene expression; however, a holistic view of pathway enrichment and inter-gene correlations is lacking. This study partly addresses this gap.

Despite rapid progress, genetics remains one of the most complex branches of medicine. The molecular mechanisms underlying many diseases, the relationships between gene expression and environmental factors, and the correlations in expression among individual genes are still not fully understood. The volume of available genetic data already exceeds our current analytical capabilities, and what has been collected represents only a small fraction of the entire genetic landscape. Due to the immense complexity of these interactions, the use of advanced bioinformatic tools is indispensable for deciphering the regulatory networks at play. By integrating microarray data with pathway enrichment analysis and visualization, our study aims to bridge the gap between clinical observation and molecular mechanism.

Our study offers new insights into the effects of maternal health status on UC-MSCs gene expression. The objective of this work was to identify specific transcriptomic signatures and signaling pathway networks (such as WNT/PCP or ECM remodeling) that underlie the molecular response of UC-MSCs to maternal diabetes and hypothyroidism.

## 2. Materials and Methods

### 2.1. Materials

The study material consisted of umbilical cords collected from women who delivered at the Clinic of Obstetrics and Pregnancy Pathology of the Independent Public Clinical Hospital No. 1 in Lublin. In total, samples from nine individuals were analyzed. The participants were divided into three groups: a control group, a group of women with hypothyroidism, and a group of women with diabetes. Basic clinical characteristics of the study participants are presented in Table 1. All pregnancies were carried to term, and all patients delivered via cesarean section.

The control group consisted of healthy, non-smoking women with uncomplicated singleton pregnancies, no history of glucose intolerance or thyroid dysfunction, who delivered healthy full-term infants. The diabetes group included patients with gestational diabetes mellitus (GDM) diagnosed by an oral glucose tolerance test (OGTT) with 75 g of glucose, according to the current guidelines of the Polish Diabetes Association [26]. The hypothyroid group included women with overt hypothyroidism, diagnosed according to the current standards of the Polish Endocrine Society [27], characterized by elevated TSH levels above the gestational reference range. All diagnoses were made by certified specialists in diabetology or endocrinology. Patients with multiple pregnancies, hypertension, or other chronic systemic diseases were excluded from the study to ensure group homogeneity.

Each participant was informed about the purpose and course of the study and provided written informed consent to participate in the project. The study was conducted with the approval of the Bioethics Committee of the Medical University of Lublin (KE-0254/7/01/2023) and carried out at the Department of Clinical Genetics of the Medical University of Lublin.

### 2.2. RNA Isolation and Microarray

In this study, UC-MSCs were isolated, cultured, and subjected to RNA extraction according to a previously described protocol [19]. Umbilical cords collected by the attending physician were transported to the Department of Clinical Genetics at the Medical University of Lublin, where they were enzymatically digested with type I collagenase to isolate UC-MSCs. The obtained cells were subsequently used to establish primary cell cultures. Successful isolation of the target cell population was confirmed by flow cytometry based on the presence of characteristic surface antigens, including CD73, CD90, and CD105.

Next, RNA was isolated from the collected cells using a modified Chomczyński and Sacchi method [24,28]. The extracted genetic material was subsequently used for gene expression profiling using microarray technology.

In total, the expression of 48,226 genes was analyzed. For this purpose, the GeneChip Human Gene 2.0 ST Array microarray system (Affymetrix, Santa Clara, CA, USA) was used. The study was conducted using RNA samples with an integrity index greater than 7.5, in accordance with the manufacturer’s instructions. Microarray scanning was performed using the Affymetrix GeneChip Scanner 3000 7G with Workstation and AutoLoader (Affymetrix, Santa Clara, CA, USA). Data from CEL files were processed using Affymetrix GeneChip Command Console Software version 3.1.4 (AGCC; Affymetrix, Santa Clara, CA, USA).

Differences in gene expression between the individual groups were analyzed using Affymetrix Transcriptome Analysis Console software version 4.0.2 (TAC; Affymetrix, Santa Clara, CA, USA). The analysis was performed in accordance with the software manufacturer’s guidelines.

### 2.3. Differential Gene Expression Analysis

Differential gene expression analyses were performed by comparing the disease groups (diabetes or hypothyroidism) with the control group. Genes were considered differentially expressed if they met the following criteria: absolute log2 fold change (|log2FC|) ≥ 1 and a false discovery rate-adjusted *p*-value (FDR) < 0.25. Differentially expressed genes (DEGs) were classified as upregulated or downregulated according to the direction of the log2FC.

### 2.4. Identification of Highly and Lowly Expressed Genes

To characterize global expression patterns across all samples, mean log2 expression values were calculated for each gene. Genes corresponding to the upper 10% of the global expression distribution were defined as highly expressed, whereas genes within the lowest expression range after background filtering were considered lowly expressed. Probes lacking valid gene symbols or corresponding to non-coding transcripts were excluded prior to analysis.

### 2.5. Pathway Enrichment Analysis

Pathway enrichment analyses were performed using Metascape (https://metascape.org (accessed on 28 November 2025)). Lists of differentially expressed genes (upregulated and downregulated), as well as globally highly and lowly expressed genes, were analyzed separately. Enrichment of Gene Ontology biological processes and pathway databases was assessed using the default statistical parameters implemented in Metascape. Statistical significance was evaluated using LogP values, defined as −log10 (*p*-value). Pathways that met the statistical significance threshold and ranked within the top 20% of enrichment scores were selected for downstream analyses.

### 2.6. Protein–Protein Interaction Network Analysis

Protein–protein interaction (PPI) networks were constructed using the STRING database (version 11.5), restricted to Homo sapiens and medium-confidence interactions. The interaction networks were exported and visualized using Cytoscape (version 3.10.4). Node color and size were mapped to log2 fold change values and network degree, respectively.

### 2.7. Heatmap Visualization

Heatmaps were generated using gene-level log2 expression values across individual samples. Expression matrices were visualized with row-wise scaling. Hierarchical clustering was performed using Ward’s linkage method and Euclidean distance. Expression levels were represented using a blue–white–red color gradient, indicating low to high expression.

## 3. Results

### 3.1. Study Participants

Genetic material from nine individuals was analyzed. The study participants were divided into three groups: a control group, a group of women with hypothyroidism, and a group of women with diabetes. Basic information regarding the health status of the examined women was collected and is presented in Table 1.

### 3.2. The Impact of Maternal Diabetes During Pregnancy on Gene Expression and Signaling Pathways in UC-MSCs

**Figure 1:**
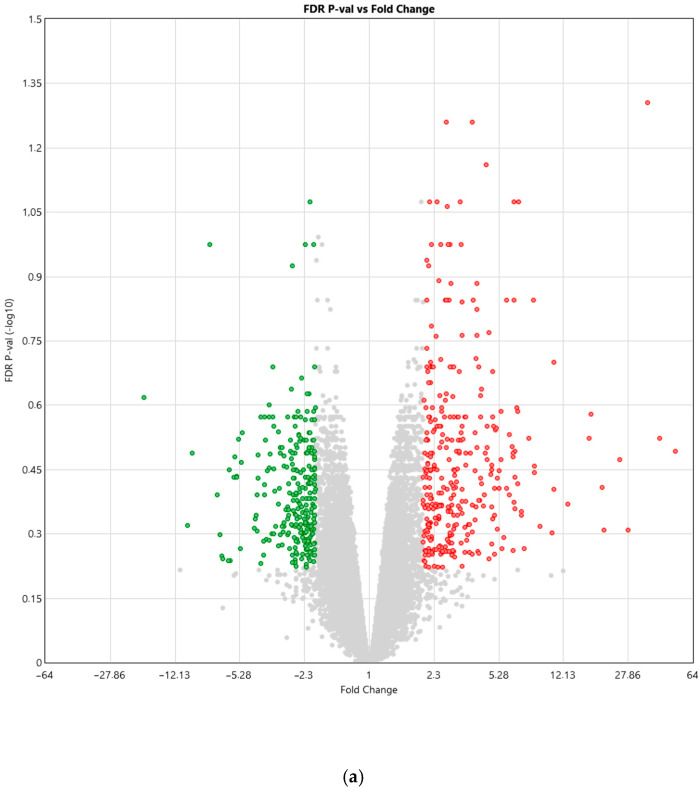

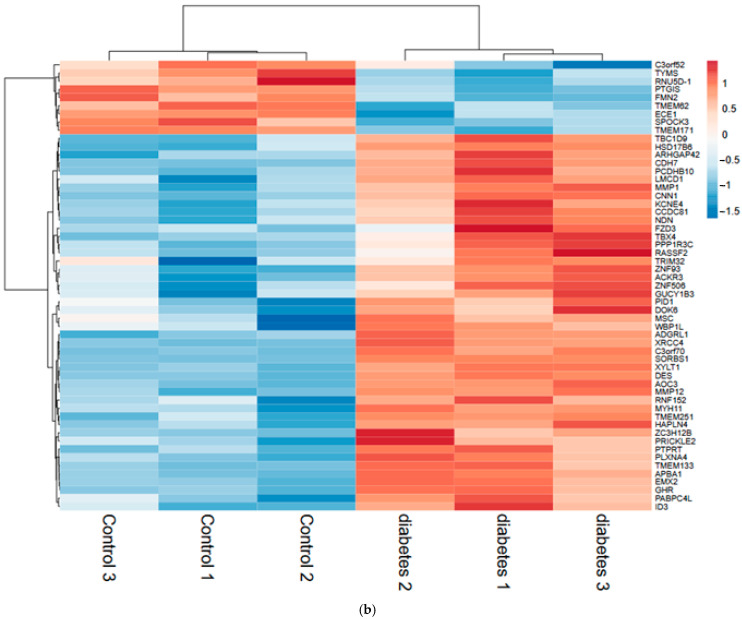
(**a**) Volcano plot illustrating the relationship between FDR-adjusted *p*-values and fold changes in the gene expression analysis of UC-MSCs derived from women with diabetes compared to UC-MSCs from the control group. Red points—genes expressed significantly more than twice in UC-MSCs from diabetic women compared to UC-MSCs from the control group (fold change: >2); green points—genes expressed significantly less than twice in UC-MSCs from diabetic women compared to UC-MSCs from the control group (fold change: <−2); gray points—genes expressed in UC-MSCs from diabetic women compared to UC-MSCs from the control group (*p* > 0.05). (**b**) Heatmap depicting differences in the expression of individual genes between the control group and the group of women with diabetes. Expression levels were represented using a blue–white–red color gradient, indicating low to high expression.

Microarray analysis revealed differential gene expression between the control group and the group of women with diabetes (Figure 1). In the diabetes group, 340 genes (0.71%) were upregulated, while 268 genes (0.56%) were downregulated compared with UC-MSCs from the control group.

Pathway enrichment analysis identified genes significantly associated with pathways enriched in the diabetes group: *MIR324*, *PTGIS*, *PTPRT*, *PID1*, *MSC*, *GHR*, *WBP1L*, *ACKR3*, *FZD3*, *CDH7*, *NDN*, *APBA1*, *PTPRT*, *TRIM32*, *XYLT1*, *PLXNA4*, *PRICKLE2*, *TBX4*, *ID3*, *PRICKLE1*, *ECE1*, *CNN1*, *MYH11*, *KCNE4*, *SORBS1*, *TRIM32*, *FMN2*, *GUCY1B1*, and *DES* (assuming (FDR) < 0.25).

**Figure 2:**
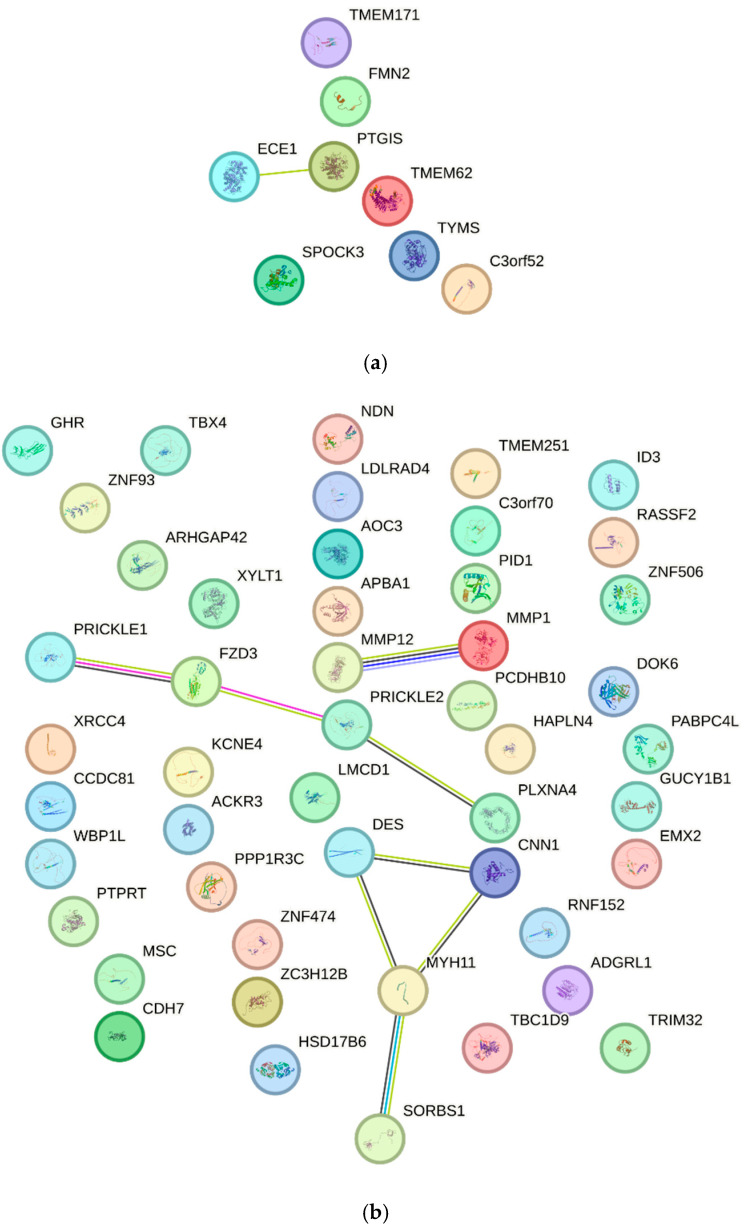
(**a**) Genes with reduced expression in MSCs derived from women with diabetes, along with their associated interactions. (**b**) Genes with increased expression in MSCs derived from the women with diabetes, along with the associated interactions.

**Figure 3:**
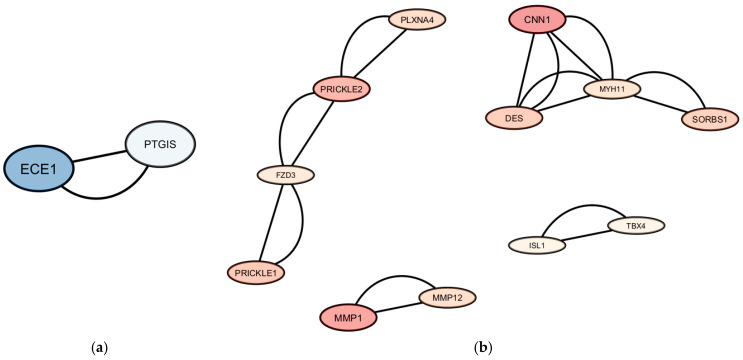
(**a**) Interaction network among genes with reduced expression in MSCs derived from women with diabetes. (**b**) Interaction network among genes with increased expression in MSCs derived from women with diabetes.

The genes—exhibiting altered expression in MSCs derived from women with diabetes—are presented in Figure 2. The relationships among genes with decreased or increased expression are illustrated in Figure 3.

Additionally, protein–protein interaction (PPI) analysis revealed that the constructed PPI network comprised 31 proteins connected by 21 interactions. The PPI enrichment *p*-value was 2.49 × 10^−7^, indicating that the network contains significantly more interactions than expected by chance. The average node degree (1.35) and clustering coefficient (0.285) suggest the presence of functional biological modules.

**Figure 4:**
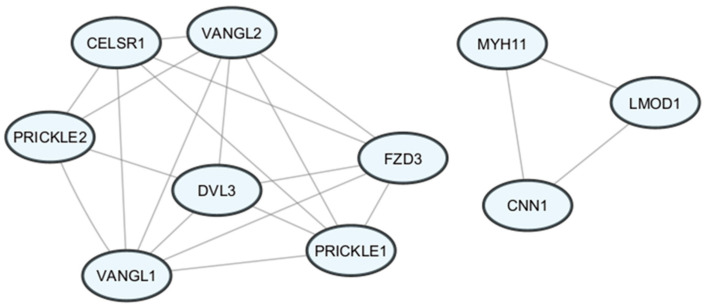
PPI network of genes significantly regulated in the diabetes group. The graph illustrates the interaction network among gene products belonging to the most enriched biological pathways identified in the microarray analysis. Connections between nodes represent high-confidence protein–protein interactions obtained from the STRING database (version 12.0). Visualization was performed using Cytoscape (version 3.10.4). The nodes form two main subnetworks: the PCP (planar cell polarity) complex, including *PRICKLE1/2*, *VANGL1/2*, *CELSR1*, *DVL3*, and *FZD3*, and a smaller module comprising *MYH11*, *CNN1*, and *LMOD1*, which are associated with smooth muscle cell function.

The most compact module comprised proteins associated with WNT/planar cell polarity (WNT/PCP) signaling, including *FZD3*, *PRICKLE1*, *PRICKLE2*, *VANGL1*, *VANGL2*, and *CELSR1*. The second module comprised genes related to smooth muscle activity, such as *MYH11*, *CNN1*, and *LMOD1* (Figure 4).

Moreover, in the diabetes group, increased activity was observed in the following pathways: Wnt signaling pathway, planar cell polarity pathway (GO:0060071), smooth muscle contraction (R-HSA-445355), autonomic nervous system development (GO:0048483), non-canonical Wnt signaling pathway (GO:0035567), Wnt signaling (WP428), actomyosin structure organization (GO:0031032), muscle contraction (R-HSA-397014), synapse assembly (GO:0007416), and neuron migration (GO:0001764) as well as decreased activity of the negative regulation of protein metabolic process pathway (GO:0051248).

### 3.3. The Impact of Maternal Hypothyroidism During Pregnancy on Gene Expression and Signaling Pathways in UC-MSCs

**Figure 5:**
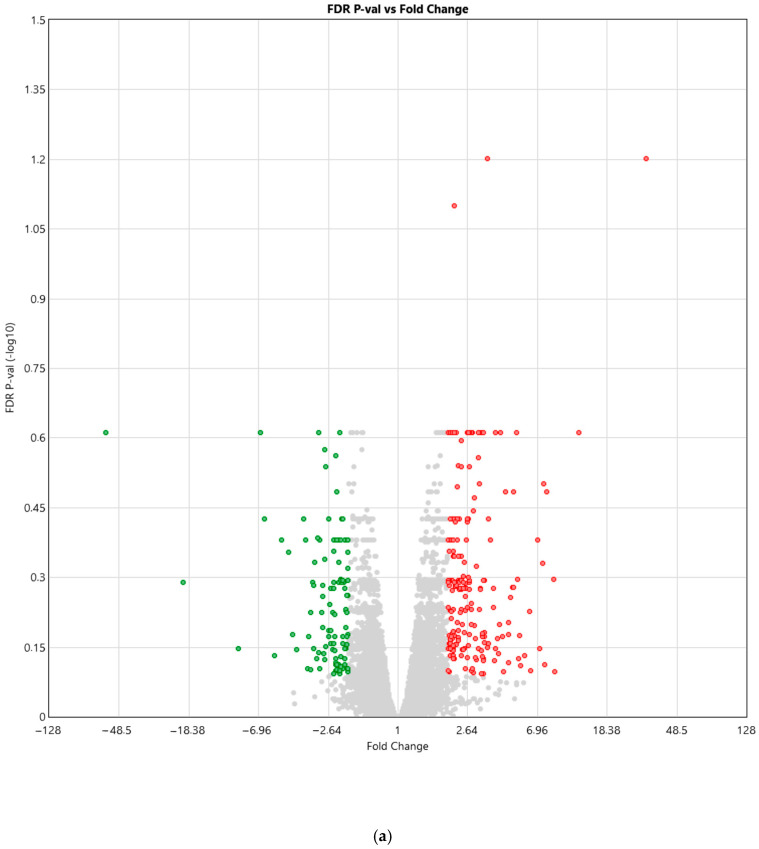

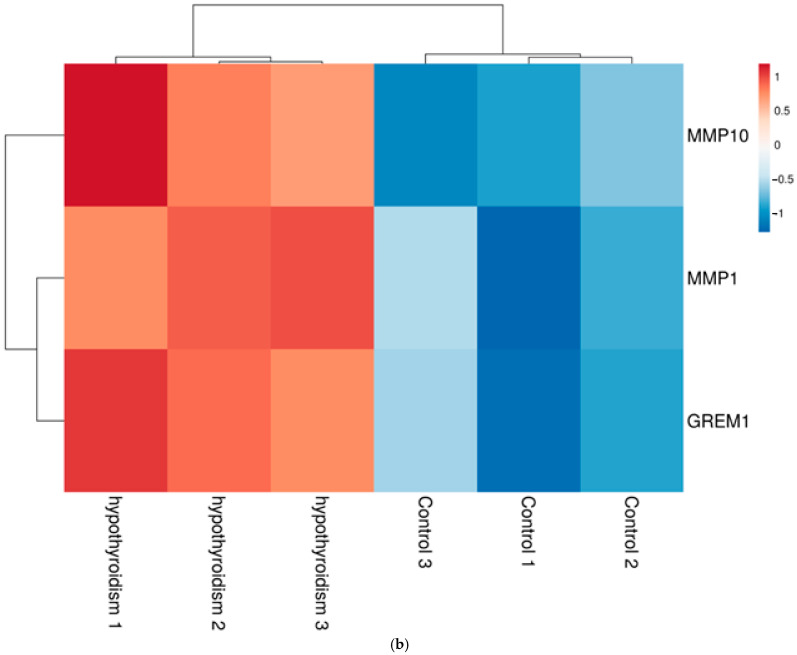
(**a**) Volcano plot showing the relationship between FDR-adjusted *p*-values and fold change in the gene expression analysis of UC-MSCs obtained from women with hypothyroidism and UC-MSCs from the control group. Red points—genes expressed significantly more than twice in UC-MSCs from diabetic women compared to UC-MSCs from the control group (fold change: >2); green points—genes expressed significantly less than twice in UC-MSCs from diabetic women compared to UC-MSCs from the control group (fold change: <−2); gray points—genes expressed in UC-MSCs from diabetic women compared to UC-MSCs from the control group (*p* > 0.05). (**b**) Heatmap illustrating differences in the expression of individual genes between the control group and the group of women with hypothyroidism. Expression levels were represented using a blue–white–red color gradient, indicating low to high expression.

Microarray analysis revealed differential gene expression between the control group and the group of women with hypothyroidism (Figure 5). It was observed that in the hypothyroidism group, 115 genes (0.43%) were upregulated, while 205 genes (0.24%) were downregulated compared to UC-MSCs from the control group (filtering criteria: fold change: >2 or <−2; *p*-value < 0.05).

**Figure 6:**
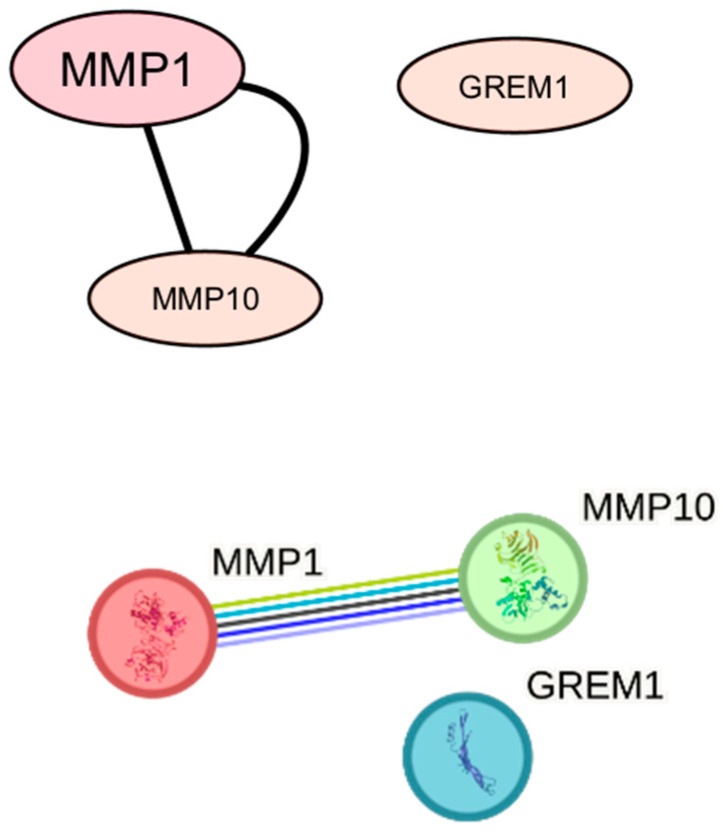
Protein–protein interaction network for genes from the enriched pathways GO:0030198 and GO:0043062 in the group of women with hypothyroidism. MMP1 and MMP10 form a direct interaction in the STRING database, whereas GREM1, despite being associated with the same pathways, does not exhibit direct connections within the analyzed network under the applied confidence criteria.

In the group of women with hypothyroidism (at an FDR threshold of <0.25), microarray analysis revealed increased expression of *MMP1*, *MMP10*, and *GREM1*, which are associated with the extracellular matrix organization (GO:0030198) and extracellular structure organization (GO:0043062) pathways (Figure 5). No genes with decreased expression met this threshold. Pathway enrichment analysis demonstrated an interaction network between MMP1 and MMP10. No direct interaction was detected between GREM1 and either MMP1 or MMP10, despite their association with the same pathways (Figure 6).

## 4. Discussion

Our study demonstrated the presence of a compact module of proteins associated with the WNT/PCP pathway in UC-MSCs from women with diabetes. The WNT/PCP pathway plays a critical role in embryogenesis. Proteins classified within this pathway participate in morphogenetic processes; regulate cellular polarization and orientation; and are involved in early stages of fetal development, including gastrulation and neurulation, as well as in later organogenesis [29]. These findings suggest that maternal diabetes during pregnancy may influence these processes by affecting genes involved in the WNT/PCP pathway in fetal UC-MSCs.

The involvement of the WNT/PCP pathway in the pathogenesis of diabetes has been previously demonstrated, and our findings corroborate this observation. This pathway contributes to the development of diabetic nephropathy, a common complication of diabetes, by inducing podocyte injury and promoting disease progression. It has been observed that the WNT/PCP is activated during renal injury in individuals with diabetes. Additionally, the WNT/PCP pathway is involved in the differentiation and proliferation of pancreatic cells, and its dysregulation contributes to the progression of diabetes [30]. Our study further confirms that diabetes affects proteins associated with the WNT/PCP pathway.

Moreover, our study indicates the presence of interrelationships among the genes *PRICKLE1/2*, *VANGL1/2*, *CELSR1*, *DVL3*, and *FZD3.* These observations are consistent with the findings reported by Wang et al., who demonstrated that *PRICKLE1* supports the activity of *VANGL2*. Their analysis revealed a network of interactions among WNT/PCP pathway genes similar to that observed in our study; however, Wang et al. reported the presence of additional genes within this complex, including *DAAM2*, *CDC42*, *ACTR3*, *TJP1*, *RHOA*, *CTNNB1*, *KIF15*, and *KIF13B.* Although these genes were included in our analysis, pathway enrichment did not indicate their statistical significance within the WNT/PCP pathway. Notably, Wang et al. used primary Sertoli cells in their study [31]. Therefore, the observed differences may be attributable to the use of different biological materials, as MSCs exhibit different plasticity than Sertoli cells, or to the limited sample size of our study.

Gong et al. demonstrated that MSCs undergoing osteogenic differentiation exhibit lower levels of VANGL2, whereas VANGL2 levels increase during adipogenic differentiation [32]. Therefore, the results of our study suggest that maternal diabetes may influence the differentiation trajectory of MSCs.

The genes *MYH11*, *CNN1*, and *LMOD1* are characteristic of smooth muscle cells, where they contribute to cytoskeletal organization and contraction. In our study, we observed interactions among these genes in UC-MSCs derived from women with diabetes. These interactions are consistent with the findings of Perisic Matic et al., who reported a positive correlation among *LMOD1*, *MYH11*, and *CNN1* in smooth muscle cells [33]. This observation is relevant to MSCs, as one of their differentiation pathways leads to muscle cells.

Additionally, our study demonstrates increased expression of the genes *MYH11*, *CNN1*, and *LMOD1* in UC-MSCs derived from women with diabetes. In studies on smooth muscle cells, it was observed that knockdown of *LMOD1* enhances cell proliferation and migration [34]. In our study, we observed an increase in *LMOD1* expression, which may suggest a reduction in cell proliferation and migration. This observation is consistent with the findings of Hickson et al., who reported decreased proliferation of MSCs derived from patients with gestational diabetes [25,35].

Perisic Matic et al. reported that in activated muscle cells, the expression of *CNN1* and *MYH11* is reduced [33]. In contrast, our study indicates that diabetes increases the expression of these genes. This alteration may contribute to reduced activation of UC-MSCs differentiating toward muscle cells.

In the group of women with hypothyroidism, our study revealed elevated expression of *MMP1*, *MMP10*, and *GREM1*, which are associated with the pathways GO:0030198 and GO:0043062. Both pathways are involved in extracellular matrix formation, which, as a network of molecules, participates in numerous cellular processes, including proliferation, differentiation, apoptosis, and intercellular signaling [35,36,37,38].

*MMP1* and *MMP10* are genes that encode matrix metalloproteinases (MMPs), which are proteins involved in the organization of the extracellular matrix. Collagen plays a central role in this process, as it constitutes the main component of connective tissue. The degradation of collagen and other proteins by MMPs regulates tissue morphogenesis. MMP1 is associated with cell proliferation and pro-inflammatory activity, whereas MMP10 inhibits proangiogenic processes [39].

Trentin et al. reported that treatment of astrocytes with T3 stimulates cell proliferation and the rearrangement of extracellular matrix proteins [40]. In our study, we observed a similar effect in UC-MSCs. Specifically, among women with hypothyroidism, we detected increased expression of *MMP1*, *MMP10*, and *GREM1.* This may be related to the administration of levothyroxine, a medication that supplements T4 deficiency, which is subsequently converted to T3. T3 may, similarly to its effects in astrocytes, stimulate the reorganization of extracellular matrix proteins.

It has been demonstrated that elevated *MMP1* expression occurs in highly migratory MSCs [41]. Therefore, MSCs derived from women with hypothyroidism may exhibit increased motility, which could enhance the therapeutic potential of these cells.

*MMPs* also participate in directing MSC differentiation. It has been shown that *MMP1* expression decreases during osteogenic differentiation [42]. This suggests that UC-MSCs derived from women with hypothyroidism may exhibit reduced plasticity with respect to this differentiation pathway.

Our study also demonstrated increased expression of *GREM1* associated with hypothyroidism. Therefore, its expression in UC-MSCs may have implications for fetal development. However, overexpression of *GREM1* has also been reported in numerous pathological conditions, including pulmonary fibrosis, diabetic nephropathy, chronic pancreatitis, and certain cancers. These findings suggest that hypothyroidism may similarly influence *GREM1* expression. Moreover, GREM1 promotes angiogenesis, which may be beneficial for tissue regeneration [43].

Our study is one of the few to simultaneously examine diabetes and hypothyroidism in the same cellular model, allowing us to identify specific changes in MSCs under the influence of maternal stress. The significant changes in gene expression patterns observed in UC-MSCs from women with pregnancies complicated by diabetes and hypothyroidism are consistent with the emerging concept of metabolic memory and maternal–fetal programming. These findings suggest that the intrauterine environment—characterized by chronic hyperglycemia or hormonal imbalance—can induce stable molecular changes in fetal stem cells that persist after the initial stimulus ends. We tentatively hypothesize that dysregulation of the WNT/PCP pathway and ECM-related genes (such as *MMP1* and *GREM1*) reflects the epigenetic imprint left by the maternal physiological state. In our previous work, where we investigated the molecular aspects of umbilical cord mesenchymal stem cells, we demonstrated that clinical factors such as the course of pregnancy and delivery, and the health status of the mother during pregnancy are important in the context of the molecular profile of MSCs, confirming that the intrauterine environment programs these cells [19,24,25,44,45,46,47]. Such programming of MSCs during a critical period of development may not only influence the long-term health trajectory of the offspring but also the functional quality and therapeutic potential of these cells if used in regenerative medicine. However, the conclusions drawn from this study should be interpreted with caution due to the small cohort size and the lack of experimental validation of the obtained results.

## 5. Strengths and Limitations

This study provides novel insights into the signaling pathways involved in the pathogenesis of diabetes and hypothyroidism. Furthermore, it confirms that maternal health influences the molecular mechanisms of UC-MSCs, which serve as a representation of fetal cells. In addition, the study demonstrates the utility of bioinformatics tools and highlights their role, particularly in the field of genetics.

Moreover, it is important to emphasize that MSCs are commonly studied in regenerative medicine. The changes we demonstrated in differentiation and migration genes suggest that stem cells collected from mothers with chronic diseases may have different therapeutic potential. This has important implications for umbilical cord biobanking. If their molecular profile is altered, their effectiveness in tissue regeneration may be different, which is another aspect of future research. Our results support the idea that UC-MSCs molecularly reflect the metabolic challenges encountered during fetal development. This study opens the door to understanding how maternal metabolic health shapes the initial molecular profile of the newborn.

On the other hand, the study was conducted on a cohort of nine individuals. The small sample size represents a limitation of this work, and the results should be validated in a larger cohort. Additionally, gene expression should be confirmed using PCR as a reference method.

Despite the limited sample size of this pilot study, stringent bioinformatics filtering methods allowed the identification of genes with high statistical significance, aiming to select promising candidates for further investigation.

## 6. Conclusions

In summary, we examined gene expression in UC-MSCs from the control group, women with hypothyroidism, and women with diabetes using microarrays. Additionally, pathway enrichment analysis allowed us to identify interaction networks among the evaluated genes.

In the diabetes group, the most compact module was formed by proteins associated with WNT/planar cell polarity (WNT/PCP) signaling, including *FZD3*, *PRICKLE1*, *PRICKLE2*, *VANGL1*, *VANGL2*, and *CELSR1*. The second module comprised genes related to smooth muscle activity, such as *MYH11*, *CNN1*, and *LMOD1.* In the hypothyroidism group, increased expression of *MMP1*, *MMP10*, and *GREM1* was observed. In this group, an association was also identified between the extracellular matrix organization (GO:0030198) and extracellular structure organization (GO:0043062) pathways.

However, it should be noted that this study represents a preliminary screening. The obtained results should be validated by PCR analysis in a larger cohort.

## Figures and Tables

**Table 1:** Characteristics of the control group, the hypothyroidism group, and the diabetes group.

Parameter	Control Group N = 3	Group with Hypothyroidism N = 3	Group with Diabetes N = 3
		Mean +/− SD	
Mother’s age	40.7 +/− 4.6	35.5 +/− 3.5	36.5 +/− 0.7
Pregnancy	2.3 +/− 1.2	1.5 +/− 0.7	3.5 +/− 2.1
Birth	1.7 +/− 0.6	1.5 +/− 0.7	3.0 +/− 2.8
HBD	38.3 +/− 0.6	39.0 +/− 0.0	38.0 +/− 1.4
Newborn’s body weight	3036.7 +/− 334.7	3070.0 +/− 410.1	2995.0 +/− 120.2
pH	7.4 +/− 0.0	7.4 +/− 0.0	7.3 +/− 0.0
pCO_2_	41.8 +/− 0.4	37.4 +/− 4.6	47.3 +/− 4.9
pO_2_	33.2 +/− 5.9	35.0 +/− 6.6	31.5 +/− 13.7
cHCO_3_	22.4 +/− 1.2	23.5 +/− 0.8	24.1 +/− 1.2
WBC	12.4 +/− 5.8	10.8 +/− 1.6	7.2 +/− 1.7
RBC	3.7 +/− 0.8	4.0 +/− 0.0	3.8 +/− 0.5
HGB	10.9 +/− 1.9	13.0 +/− 0.5	11.8 +/− 0.9
HCT	32.5 +/− 5.9	36.6 +/− 0.4	33.3 +/− 2.4
MCH	29.9 +/− 4.3	32.0 +/− 1.1	31.1 +/− 1.4
MCV	88.6 +/− 9.5	90.4 +/− 1.3	88.2 +/− 4.5
MCHC	33.6 +/− 1.3	35.4 +/− 1.7	35.3 +/− 0.2
PLT	176.0 +/− 46.2	205.5 +/− 16.3	221.0 +/− 56.6

## Data Availability

The raw data supporting the conclusions of this article will be made available by the authors on request.

## Abbreviations

The following abbreviations are used in this manuscript:

MSCs	Mesenchymal stem cells
UC-MSCs	Umbilical cord-derived mesenchymal stem cells
RNA	Ribonucleic acid
WNT/PCP	WNT/planar cell polarity
*MMP1*	Matrix metallopeptidase 1
*MMP10*	Matrix metallopeptidase 10
*GREM1*	Gremlin 1
PCR	Polymerase chain reaction
UC	Umbilical cord
*MIR324*	MicroRNA 324
*PTGIS*	Prostaglandin I2 synthase
*PID1*	Phosphotyrosine interaction domain containing 1
*MSC*	Musculin
*GHR*	Growth hormone receptor
*WBP1L*	WW domain binding protein 1 like
*ACKR3*	Atypical chemokine receptor 3
*FZD3*	Frizzled class receptor 3
*CDH7*	Cadherin 7
*NDN*	Necdin, MAGE family member
*APBA1*	Amyloid beta precursor protein binding family A member 1
*PTPRT*	Protein tyrosine phosphatase receptor type T
*XYLT1*	Xylosyltransferase 1
*PLXNA4*	Plexin A4
*PRICKLE2*	Prickle planar cell polarity protein 2
*TBX4*	T-box transcription factor 4
*ID3*	Inhibitor of DNA binding 3
*PRICKLE1*	Prickle planar cell polarity protein 1
*ECE1*	Endothelin converting enzyme 1
*CNN1*	Calponin 1
*MYH11*	Myosin heavy chain 11
*KCNE4*	Potassium voltage-gated channel subfamily E regulatory subunit 4
*SORBS1*	Sorbin and SH3 domain containing 1
*TRIM32*	Tripartite motif containing 32
*FMN2*	Formin 2
*GUCY1B1*	Guanylate cyclase 1 soluble subunit beta 1
*DES*	Desmin
*PPI*	Protein–protein interaction
*VANGL1*	VANGL planar cell polarity protein 1
*VANGL2*	VANGL planar cell polarity protein 2
*CELSR1*	Cadherin EGF LAG seven-pass G-type receptor 1
*LMOD1*	Leiomodin 1
GO:0060071	Planar cell polarity pathway (Wnt signaling)
R-HSA-445355	Smooth muscle contraction signaling pathway
GO:0048483	Autonomic nervous system development signaling pathway
GO:0035567	Non-canonical Wnt signaling pathway
WP428	Wnt signaling
GO:0031032	Actomyosin structure organization signaling pathway
R-HSA-397014	Muscle contraction signaling pathway
GO:0007416	Synapse assembly signaling pathway
GO:0001764	Neuron migration signaling pathway
GO:0051248	Negative regulation of protein metabolic process signaling pathway
GO:0030198	Extracellular matrix organization signaling pathway
GO:0043062	Extracellular structure organization signaling pathway
*DAAM2*	Disheveled associated activator of morphogenesis 2
*CDC42*	Cell division cycle 42
*ACTR3*	Actin-related protein 3
*TJP1*	Tight junction protein 1
*RHOA*	Ras homolog family member A
*CTNNB1*	Catenin beta 1
*KIF15*	Kinesin family member 15
*KIF13B*	Kinesin family member 13B
MMPs	Matrix metalloproteinases
T3	Triiodothyronine
T4	Thyroxine

## References

[1] Butureanu T.A. (2025). The Pathophysiology of Wharton’s Jelly and Its Impact on Fetal and Neonatal Outcomes: A Comprehensive Literature Review. Med. Sci..

[2] Di Naro E., Ghezzi F., Raio L., Franchi M., D’Addario V. (2001). Umbilical cord morphology and pregnancy outcome. Eur. J. Obstet. Gynecol. Reprod. Biol..

[3] Lee S.M., Kim D.Y., Cho S., Noh S.M., Park H.L., Lee G. (2020). Correlations between the Status of the Umbilical Cord and Neonatal Health Status. Child Health Nurs. Res..

[4] Dubetskyi B.I., Makarchuk O.M., Zhurakivska O.Y., Rymarchuk M.I., Andriets O.A., Lenchuk T.L., Delva K.M., Piron-Dumitrascu M., Bakun O.V. (2023). Pregnancy and umbilical cord pathology: Structural and functional parameters of the umbilical cord. J. Med. Life.

[5] Alatyyat S.M., Alasmari H.M., Aleid O.A., Abdel-Maksoud M.S., Elsherbiny N. (2020). Umbilical cord stem cells: Background, processing and applications. Tissue Cell.

[6] Samsonraj R.M., Raghunath M., Nurcombe V., Hui J.H., Wijnen A.J.V., Cool S.M. (2017). Concise Review: Multifaceted Characterization of Human Mesenchymal Stem Cells for Use in Regenerative Medicine. Stem Cells Transl. Med..

[7] Yaghoubi Y., Movassaghpour A., Zamani M., Talebi M., Mehdizadeh A., Yousefi M. (2019). Human umbilical cord mesenchymal stem cells derived-exosomes in diseases treatment. Life Sci..

[8] Wang B., Jia H., Zhang B., Wang J., Ji C., Zhu X., Yan Y., Yin L., Yu J., Qian H. (2017). Pre-incubation with hucMSC-exosomes prevents cisplatin-induced nephrotoxicity by activating autophagy. Stem Cell Res. Ther..

[9] Zhang X., Liu J., Yu B., Ma F., Ren X., Li X. (2018). Effects of mesenchymal stem cells and their exosomes on the healing of large and refractory macular holes. Graefes Arch. Clin. Exp. Ophthalmol..

[10] Ding M., Shen Y., Wang P., Xie Z., Xu S., Zhu Z., Wang Y., Lyu Y., Wang D., Xu L. (2018). Exosomes Isolated From Human Umbilical Cord Mesenchymal Stem Cells Alleviate Neuroinflammation and Reduce Amyloid-Beta Deposition by Modulating Microglial Activation in Alzheimer’s Disease. Neurochem. Res..

[11] Sun G., Li G., Li D., Huang W., Zhang R., Zhang H., Duan Y., Wang B. (2018). hucMSC derived exosomes promote functional recovery in spinal cord injury mice via attenuating inflammation. Mater. Sci. Eng. C.

[12] Zhao Y., Sun X., Cao W., Ma J., Sun L., Qian H., Zhu W., Xu W. (2015). Exosomes Derived from Human Umbilical Cord Mesenchymal Stem Cells Relieve Acute Myocardial Ischemic Injury. Stem Cells Int..

[13] Sun Y., Shi H., Yin S., Ji C., Zhang X., Zhang B., Wu P., Shi Y., Mao F., Yan Y. (2018). Human Mesenchymal Stem Cell Derived Exosomes Alleviate Type 2 Diabetes Mellitus by Reversing Peripheral Insulin Resistance and Relieving β-Cell Destruction. ACS Nano.

[14] Rahbaran M., Zekiy A.O., Bahramali M., Jahangir M., Mardasi M., Sakhaei D., Thangavelu L., Shomali N., Zamani M., Mohammadi A. (2022). Therapeutic utility of mesenchymal stromal cell (MSC)-based approaches in chronic neurodegeneration: A glimpse into underlying mechanisms, current status, and prospects. Cell Mol. Biol. Lett..

[15] Shen Z., Huang W., Liu J., Tian J., Wang S., Rui K. (2021). Effects of Mesenchymal Stem Cell-Derived Exosomes on Autoimmune Diseases. Front. Immunol..

[16] Lancaster C.L., Moberg K.H., Corbett A.H. (2025). Post-Transcriptional Regulation of Gene Expression and the Intricate Life of Eukaryotic MRNAS. WIREs RNA.

[17] Kusnadi E.P., Timpone C., Topisirovic I., Larsson O., Furic L. (2022). Regulation of gene expression via translational buffering. Biochim. Biophys. Acta BBA Mol. Cell Res..

[18] Kitsiou-Tzeli S., Tzetis M. (2017). Maternal epigenetics and fetal and neonatal growth. Curr. Opin. Endocrinol. Diabetes Obes..

[19] Ozygała A., Wojciechowska G., Traczyk K., Przywara D., Petniak A., Szymanowski R., Wilińska A., Krzyżanowski A., Kwaśniewska A., Płachno B.J. (2023). Influence of Umbilical Cord Blood Biochemical Parameters and Disease Condition on the Expression of the TSG-6 Gene in Umbilical Mesenchymal Stem Cells. Med. Sci. Monit..

[20] Gong L.L., Liu H., Liu L.H. (2016). Relationship between hypothyroidism and the incidence of gestational diabetes: A meta-analysis. Taiwan J. Obstet. Gynecol..

[21] Abadi K.K., Jama A.H., Legesse A.Y., Gebremichael A.K. (2023). Prevalence of Hypothyroidism in Pregnancy and Its Associations with Adverse Pregnancy Outcomes Among Pregnant Women in A General Hospital: A Cross Sectional Study. Int. J. Womens Health.

[22] Luo J., Wang X., Yuan L., Guo L. (2021). Association of thyroid disorders with gestational diabetes mellitus: A meta-analysis. Endocrine.

[23] Karkia R., Giacchino T., Shah S., Gough A., Ramadan G., Akolekar R. (2023). Gestational Diabetes Mellitus: Association with Maternal and Neonatal Complications. Medicina.

[24] Bieńko K., Leszcz M., Więckowska M., Białek J., Petniak A., Szymanowski R., Wilińska A., Piszcz B., Krzyżanowski A., Kwaśniewska A. (2023). VEGF Expression in Umbilical Cord MSC Depends on the Patient’s Health, the Week of Pregnancy in Which the Delivery Took Place, and the Body Weight of the Newborn—Preliminary Report. Stem Cells Cloning.

[25] Przywara D., Petniak A., Gil-Kulik P. (2024). Optimizing Mesenchymal Stem Cells for Regenerative Medicine: Influence of Diabetes, Obesity, Autoimmune, and Inflammatory Conditions on Therapeutic Efficacy: A Review. Med. Sci. Monit..

[26] Araszkiewicz A., Borys S., Broncel M. (2025). Clinical recommendations for the management of people with diabetes—2025. Position of the Polish Diabetes Association. [Zalecenia kliniczne dotyczące postępowania u osób z cukrzycą—2025. Stanowisko Polskiego Towarzystwa Diabetologicznego]. Curr. Top. Diabet..

[27] Hubalewska-Dydejczyk A., Trofimiuk-Müldner M., Ruchala M., Lewiński A., Bednarczuk T., Zgliczyński W., Syrenicz A., Kos-Kudla B., Jarząb B., Gietka-Czernel M. (2021). Thyroid diseases in pregnancy: Guidelines of the Polish Society of Endocrinology [Choroby tarczycy w ciąży: Zalecenia postępowania Polskiego Towarzystwa Endokrynologicznego]. Endokrynol. Pol..

[28] Chomczynski P., Sacchi N. (1987). Single-step method of RNA isolation by acid guanidinium thiocyanate-phenol-chloroform extraction. Anal. Biochem..

[29] Shi D.L. (2022). Wnt/planar cell polarity signaling controls morphogenetic movements of gastrulation and neural tube closure. Cell Mol. Life Sci..

[30] Wang H., Zhang R., Wu X., Chen Y., Ji W., Wang J., Zhang Y., Xia Y., Tang Y., Yuan J. (2022). The Wnt Signaling Pathway in Diabetic Nephropathy. Front. Cell Dev. Biol..

[31] Wang L., Bu T., Gao S., Yun D., Chen H., Cheng C.Y., Sun F. (2025). PCP protein Prickle 1 regulates Sertoli cell and testis function via cytoskeletal organization through the recruitment of multiple regulatory proteins. Am. J. Physiol.-Cell Physiol..

[32] Gong Y., Li Z., Zou S., Deng D., Lai P., Hu H., Yao Y., Hu L., Zhang S., Li K. (2021). Vangl2 limits chaperone-mediated autophagy to balance osteogenic differentiation in mesenchymal stem cells. Dev. Cell.

[33] Perisic Matic L., Rykaczewska U., Razuvaev A., Sabater-Lleal M., Lengquist M., Miller C.L., Ericsson I., Röhl S., Kronqvist M., Aldi S. (2016). Phenotypic Modulation of Smooth Muscle Cells in Atherosclerosis Is Associated with Downregulation of *LMOD1*, *SYNPO2*, *PDLIM7*, *PLN*, and *SYNM*. Arterioscler. Thromb. Vasc. Biol..

[34] Wong D., Turner A.W., Miller C.L. (2019). Genetic Insights into Smooth Muscle Cell Contributions to Coronary Artery Disease. Arterioscler. Thromb. Vasc. Biol..

[35] Hickson L.J., Eirin A., Conley S.M., Taner T., Bian X., Saad A., Herrmann S.M., Mehta R.A., McKenzie T.J., Kellogg T.A. (2021). Diabetic Kidney Disease Alters the Transcriptome and Function of Human Adipose-Derived Mesenchymal Stromal Cells but Maintains Immunomodulatory and Paracrine Activities Important for Renal Repair. Diabetes.

[36] Cheong S., Peng Y., Lu F., He Y. (2025). Structural extracellular matrix-mediated molecular signaling in wound repair and tissue regeneration. Biochimie.

[37] Zhao T., Huang Y., Zhu J., Qin Y., Wu H., Yu J., Zhai Q., Li S., Qin X., Wang D. (2025). Extracellular Matrix Signaling Cues: Biological Functions, Diseases, and Therapeutic Targets. MedComm.

[38] Dzobo K., Dandara C. (2023). The Extracellular Matrix: Its Composition, Function, Remodeling, and Role in Tumorigenesis. Biomimetics.

[39] Jabłońska-Trypuć A., Matejczyk M., Rosochacki S. (2016). Matrix metalloproteinases (MMPs), the main extracellular matrix (ECM) enzymes in collagen degradation, as a target for anticancer drugs. J. Enzym. Inhib. Med. Chem..

[40] Gonçalves Trentin A., De Aguiar C.B.N.M., Castilho Garcez R., Alvarez-Silva M. (2003). Thyroid hormone modulates the extracellular matrix organization and expression in cerebellar astrocyte: Effects on astrocyte adhesion. Glia.

[41] Ho I.A., Chan K.Y., Ng W.H., Guo C.M., Hui K.M., Cheang P., Lam P.Y. (2009). Matrix Metalloproteinase 1 Is Necessary for the Migration of Human Bone Marrow-Derived Mesenchymal Stem Cells Toward Human Glioma. Stem Cells.

[42] Almalki S.G., Agrawal D.K. (2016). Effects of matrix metalloproteinases on the fate of mesenchymal stem cells. Stem Cell Res. Ther..

[43] Gao Z., Gao Y., Dutton L.R., Todd G., Gipson G.R., Browne C., Kerr E.M., Daly C., Plouffe B., Dunne P.D. (2025). Identification of a novel GREMLIN1 uptake pathway in epithelial cells that requires BMP binding. J. Biol. Chem..

[44] Gil-Kulik P., Krzyżanowski A., Dudzińska E., Karwat J., Chomik P., Świstowska M., Kondracka A., Kwaśniewska A., Cioch M., Jojczuk M. (2019). Potential Involvement of BIRC5 in Maintaining Pluripotency and Cell Differentiation of Human Stem Cells. Oxid. Med. Cell Longev..

[45] Gil-Kulik P., Świstowska M., Krzyżanowski A., Petniak A., Kwaśniewska A., Płachno B.J., Galkowski D., Bogucka-Kocka A., Kocki J. (2022). Evaluation of the Impact of Pregnancy-Associated Factors on the Quality of Wharton’s Jelly-Derived Stem Cells Using SOX2 Gene Expression as a Marker. Int. J. Mol. Sci..

[46] Gil-Kulik P., Chomik P., Krzyżanowski A., Radzikowska-Büchner E., Maciejewski R., Kwaśniewska A., Rahnama M., Kocki J. (2019). Influence of the Type of Delivery, Use of Oxytocin, and Maternal Age on POU5F1 Gene Expression in Stem Cells Derived from Wharton’s Jelly Within the Umbilical Cord. Oxid. Med. Cell Longev..

[47] Gil-Kulik P., Świstowska M., Kondracka A., Chomik P., Krzyżanowski A., Kwaśniewska A., Rahnama M., Kocki J. (2020). Increased Expression of BIRC2, BIRC3, and BIRC5 from the IAP Family in Mesenchymal Stem Cells of the Umbilical Cord Wharton’s Jelly (WJSC) in Younger Women Giving Birth Naturally. Oxid. Med. Cell Longev..

